# Association of Opioid and Stimulant Use Disorder Diagnoses With Fatal and Nonfatal Overdose Among People With a History of Incarceration

**DOI:** 10.1001/jamanetworkopen.2022.43653

**Published:** 2022-11-23

**Authors:** Heather Palis, Wenqi Gan, Chloe Xavier, Roshni Desai, Marnie Scow, Kali-olt Sedgemore, Leigh Greiner, Tonia Nicholls, Amanda Slaunwhite

**Affiliations:** 1 BC Centre for Disease Control, Vancouver, British Columbia, Canada; 2 Department of Psychiatry, University of British Columbia, Vancouver, British Columbia, Canada; 3 Department of Public Health Sciences, University of Connecticut School of Medicine, Farmington; 4 School of Population and Public Health, University of British Columbia, Vancouver, British Columbia, Canada; 5 Coalition of Peers Dismantling the Drug War, Vancouver, British Columbia, Canada; 6 BC Corrections, Victoria, British Columbia, Canada; 7 BC Mental Health and Substance Use Services, Vancouver, British Columbia, Canada

## Abstract

**Importance:**

Studies have suggested a rise in opioid- and stimulant-involved overdoses in recent years in North America. This risk may be acute for individuals who have had contact with the criminal justice system, who are particularly vulnerable to overdose risk.

**Objective:**

To examine the association of opioid and/or stimulant use disorder diagnoses with overdose (fatal and nonfatal) among people with histories of incarceration.

**Design, Setting, and Participants:**

In this cohort study, population-based health and corrections data were retrieved from the British Columbia Provincial Overdose Cohort, which contains a 20% random sample of residents of British Columbia. The analysis included all people in the 20% random sample who had a history of incarceration between January 1, 2010, and December 31, 2014. Outcomes were derived from 5-years of follow-up data (January 1, 2015, to December 31, 2019). Statistical analysis took place from January 2022 to June 2022.

**Exposures:**

Substance use disorder diagnosis type (ie, opioid use disorder, stimulant use disorder, both, or neither), sociodemographic, health, and incarceration characteristics.

**Main Outcomes and Measures:**

Hazard ratios (HRs) are reported from an Andersen-Gill model for recurrent nonfatal overdose events and from a Fine and Gray competing risk model for fatal overdose events.

**Results:**

The study identified 6816 people (5980 male [87.7%]; 2820 aged <30 years [41.4%]) with histories of incarceration. Of these, 293 (4.3%) had opioid use disorder only, 395 (6.8%) had stimulant use disorder only, and 281 (4.1%) had both diagnoses. During follow-up, 1655 people experienced 4026 overdoses including 3781 (93.9%) nonfatal overdoses, and 245 (6.1%) fatal overdoses. In adjusted analyses, the hazard of both fatal (HR, 2.39; 95% CI, 1.48-3.86) and nonfatal (HR, 2.45; 95% CI, 1.94-3.11) overdose was highest in the group with both opioid and stimulant use disorder diagnoses.

**Conclusions and Relevance:**

This cohort study of people with a history of incarceration found an elevated hazard of fatal and nonfatal overdose among people with both opioid and stimulant use disorder diagnoses. This study suggests an urgent need to address the service needs of individuals who have had contact with the criminal justice system and who co-use opioids and stimulants.

## Key Points

**Question:**

What is the association between opioid and/or stimulant use disorder diagnoses and fatal and nonfatal overdose among people with a history of incarceration?

**Findings:**

In this cohort study of 6816 individuals with a history of incarceration, people with both opioid and stimulant use disorder diagnoses had approximately 2.5 times the hazard of overdose compared with people with no substance use disorder diagnoses. Stimulant use disorder alone was associated with a similar hazard of fatal overdose as opioid use disorder alone.

**Meaning:**

These findings suggest that in the context of an ongoing overdose public health emergency that disproportionally impacts people with histories of incarceration, there is an urgent need for attention to the service needs of individuals who have had contact with the criminal justice system who co-use opioids and stimulants.

## Teaser

This cohort study examines the association of opioid use disorder and stimulant use disorders diagnoses with fatal and nonfatal overdose among people with histories of incarceration in British Columbia.

## Introduction

North America is facing an unprecedented rise in illicit drug toxicity (overdose) deaths. While national in scope, British Columbia (BC) (Canada’s westernmost and third most populous province) has consistently reported disproportionately higher rates of illicit drug toxicity deaths compared with national rates.^1^ This rise in deaths was first deemed a public health emergency in April 2016, when mortality rates began increasing due to the contamination of the illicit drug supply with fentanyl (a potent opioid) and its analogs, which was detected in only 5% of deaths in 2012, and 85% of deaths in 2020.^2^ Alongside this rise, methamphetamine detection has grown from 14% to 44%, and cocaine and methamphetamine have remained the second and third most commonly detected substances in illicit drug toxicity deaths.^2^

Recent studies^3,4^ have indicated a rise in stimulant use among people who use opioids and among people with opioid use disorder (OUD) diagnoses, and have referred to this rise in concurrent use of opioids and stimulants as twin epidemics. Studies of justice-involved populations (ie, individuals who are involved in the criminal justice system) in Canada and the United States have similarly identified a rise in polysubstance use in recent years among people who use opioids^5,6,7^ and concurrent stimulant use is a known risk factor for overdose in this population.^7,8^ For example, a study^8^ of polysubstance use profiles in formerly incarcerated people in Kentucky found that concurrent opioid and stimulant use was associated with higher overdose risk, compared with all other polysubstance use patterns. A study^7^ in Washington state identified cocaine as the most commonly detected substance found alongside opioids in opioid-related overdose deaths among people released from prison.

In BC, studies have consistently identified that people who have been incarcerated face an elevated risk of overdose. For example, a recent study^9^ identified that people with an incarceration history in BC were 4 times more likely than the general population to die of overdose. Furthermore, the risk of nonfatal overdose (NFOD) is elevated in the weeks immediately following release^10^ and is estimated to occur at least 20 to 30 times more often than fatal overdose.^11^ As rates of fatal and NFOD climb in BC alongside a rise in methamphetamine use,^5^ and disproportionately affect justice-involved populations, interventions have been almost exclusively focused on opioid use, and offered for people with OUD.^12^

While opioid and stimulant polysubstance use has been well-established as a risk factor for overdose, the extent to which people with OUD and/or stimulant use disorder (STUD) face compounded overdose risk relative to people with neither disorder has not been quantified. In the context of a continued focus on opioids as a primary driver of overdose deaths, this analysis will provide critical information about the OUD and STUD profiles of justice-involved people who are experiencing the highest risk of fatal and nonfatal overdose in BC. Findings can be used to inform the design of interventions to best meet the substance use service needs of justice-involved populations with OUD and/or STUD in both correctional and community settings.

## Methods

This cohort study followed the Strengthening the Reporting of Observational Studies in Epidemiology (STROBE) reporting guideline. The Provincial Overdose Cohort was assembled under section 52(2) of British Columbia’s Public Health Act in response to the 2016 declaration of overdose as a public health emergency in BC. This analysis was conducted using Provincial Overdose Cohort data as part of the Britsh Columbia Centre for Disease Control’s public health functions and therefore institutional ethical approval and informed consent was not required.

### Study Design

This study used a cohort design based on a 20% random sample of the general population in BC contained within the Provincial Overdose Cohort.^13^ People with a record of release from a provincial correctional center (January 1, 2010, to December 31, 2014) were identified using provincial incarceration records. The presence or absence of diagnosis of OUD or STUD at baseline was identified in this sample using hospital admissions and outpatient data. Six people had missing data on sex and were excluded, leaving 6816 people. Participants were classified into 4 exposure groups: (1) no OUD or STUD; (2) OUD only; (3) STUD only; (4) OUD and STUD. The cohort was followed up with for 5 years using linked administrative health data to identify people who experienced fatal and NFOD events.

### Outcome Measures

The overdose algorithm (eTable 1 in the Supplement) makes use of data from poison control, ambulance, emergency department, hospital, and health care provider billing records and has been previously published.^10,14^ Fatal overdoses are those for which a record of accidental or undetermined illicit drug toxicity was available in the BC Coroners Service records, or where the date of death overlapped with the date of an overdose episode. All other overdose events were classified as nonfatal. Cases where records from various sources were separated by less than 24 hours were treated as a single unique overdose record.

### Exposure Measures

The main exposure of interest was substance use disorder (SUD) diagnosis: (1) No OUD or STUD; (2) OUD only; (3) STUD only; (4) OUD and STUD (eTable 2 in the Supplement). SUD diagnoses were determined using hospital (*International Classification of Diseases, Ninth Revision* [*ICD-9*]) and primary care (*International Statistical Classification of Diseases and Related Health Problems, Tenth Revision* [*ICD-10*]) records for STUD (*ICD-9* = 304.2, 304.4, 305.6, 305.7; *ICD-10* = F14, F15) and OUD (*ICD-9* = 304.0, 304.7, 305.5; *ICD-10* = F11), between January 1, 2010, to December 31, 2014. Other exposures determined at baseline were: age, sex, Elixhauser comorbidity index^15^ (eTable 3 in the Supplement), co-occurring mental illness diagnosis (eTable 4 in the Supplement), number of prior incarcerations, and region of residence.

### Statistical Analysis

Baseline demographic and health characteristics were compared across the four groups using χ^2^ tests or Fisher exact tests when there was an expected value less than 5. Person-time of follow-up was determined for each person from the baseline date (January 1, 2015) to the date of fatal or NFOD event, the date of death from other causes, or the end of follow-up (December 31, 2019). For people who were incarcerated during follow-up, follow-up ended on the date of incarceration and was restarted on the date of release. Because people could experience multiple NFODs, NFODs were treated as recurrent events. Follow-up was ended on the date of a NFOD, restarted the next day, and then ended on the next NFOD; person-time was calculated using each pair of start and end dates.

The incidence rate was calculated using the number of cases divided by total person-years at risk, and the 95% CI for the incidence rate was estimated using an exact method under the assumption of a Poisson distribution. Hazard ratios (HR) and 95% CIs were calculated for NFOD events using the Andersen-Gill model^16^ and for fatal overdose events using the Fine and Gray competing risk model.^17^ The Fine and Gray competing risk model took into account the deaths from other causes that precluded the possibility of overdose death. If a 95% CI did not contain the value 0, the *P* value was <.05. In sensitivity analysis, a Cox proportional-hazards model was used to calculate HRs for fatal overdose.^18^ Stratified analyses were conducted by age and sex to examine effect modification of these variables on the relationship between SUD and fatal and NFOD. Statistical significance for interaction terms was examined in the adjusted models. All statistical tests were 2-sided, and were performed using R version 3.5.2 (R Project for Statistical Computing). Statistical analysis took place from January 2022 to June 2022.

## Results

The sample included 6816 people (5980 male [87.7%]; 2820 aged <30 years [41.4%]) with at least 1 release from a provincial correctional center between January 1, 2010, and December 31, 2014. The total study follow-up time was 30 861.3 person-years; with a mean (SD) of 4.5 (0.9) person-years per person. Of the 6816 people, 5847 (85.8%) did not have a diagnosis of OUD or STUD at baseline, 293 (4.3%) had a diagnosis of OUD only, 395 (6.8%) had a diagnosis of STUD only, and 281 (4.1%) had a diagnosis of both STUD and OUD (Table 1).

**Table 1.** Baseline Characteristics by SUD Diagnosis^a^

Characteristic	No. (%)				*P* value
	No OUD or STUD (n = 5847)	OUD only (n = 293)	STUD only (n = 395)	OUD and STUD (n = 281)	
Age group, y					<.001
<30	2400 (41.0)	127 (43.3)	180 (45.6)	113 (40.2)	
30-39	1659 (28.4)	94 (32.1)	124 (31.4)	102 (36.3)	
≥40	1788 (30.6)	72 (24.6)	91 (23.0)	66 (23.5)	
Sex					<.001
Male	5256 (89.9)	226 (77.1)	309 (78.2)	189 (67.3)	
Female	591 (10.1)	67 (22.9)	86 (21.8)	92 (32.7)	
Mental illness diagnosis^b^					<.001
Yes	2039 (34.9)	207 (70.6)	318 (80.5)	244 (86.8)	
No	3808 (65.1)	86 (29.4)	77 (19.5)	37 (13.2)	
Elixhauser comorbidity index^c^					<.001
None	5458 (93.3)	235 (80.2)	295 (74.7)	166 (59.1)	
1	243 (4.2)	42 (14.3)	63 (15.9)	71 (25.3)	
≥2	146 (2.5)	16 (5.5)	37 (9.4)	44 (15.7)	
No. of prior incarcerations^d^					<.001
1	2763 (47.3)	76 (25.9)	125 (31.6)	87 (31.0)	
2	1084 (18.5)	58 (19.8)	80 (20.3)	36 (12.8)	
3-4	1027 (17.6)	66 (22.5)	80 (20.3)	60 (21.4)	
≥5^e^	973 (16.6)	93 (31.7)	110 (27.8)	98 (34.9)	
Region of residence					<.001
Interior	748 (12.8)	36 (12.3)	50 (12.7)	25 (8.9)	
Fraser	1789 (30.6)	110 (37.5)	114 (28.9)	91 (32.4)	
Vancouver Coastal	955 (16.3)	48 (16.4)	119 (30.1)	131 (46.6)	
Island	813 (13.9)	57 (19.5)	47 (11.9)	13 (4.6)	
Northern	562 (9.6)	13 (4.4)	59 (14.9)	17 (6.0)	
Unspecified^f^	980 (16.8)	29 (9.9)	6 (1.5)	4 (1.4)	

Abbreviations: OUD, opioid use disorder; STUD, stimulant use disorder; SUD, substance use disorder.

^a^ Data are presented as the number of people (column proportion, %).

^b^ Mental illness includes anxiety, depression, or stress or adjustment disorder.

^c^ Excluding mental illness and substance use categories.

^d^ Occurred between 2010 and 2014.

^e^ Median, 2; mean, 2.9; range, 1-35; IQR, 1-4.

^f^ 1019 (15%) people had missing data on residential postal codes at baseline (2014), and therefore their region of residence could not be identified. Because the proportion is not small, they are reflected in an unspecified group so that persons with missing data on this variable could remain in the analysis. There was no missing data on other variables.

During follow-up, 1655 people (24.3%) experienced 4026 overdose events including 3781 (93.9%) NFOD events, and 245 (6.1%) fatal events. Of the 1504 people who experienced a NFOD, 757 (50.3%) had more than 1 NFOD (median [range], 3 [2-21]; IQR, 2-4). Of the 245 people who experienced fatal overdose events, 94 (38.3%) experienced at least 1 prior NFOD during follow-up. Of the 20% random sample of individuals who were released from incarceration during the study, 281 (4.1%) had OUD and STUD diagnoses; however, they made up 566 (15.0%) of NFOD events, and 28 (11.4%) of fatal events.

The crude incidence rate of NFOD events during follow-up was 122.5 (95% CI, 118.6-126.5) per 1000 person-years over the entire study sample. The crude overdose mortality rate was 7.9 (95% CI, 7.0-9.0) per 1000 person years. The incidence of NFOD was lowest in the no OUD or STUD group, higher for the STUD only group, higher in the OUD only group, and highest in the OUD and STUD group. Overdose mortality was highest in the OUD and STUD group and was nearly identical in the OUD only and STUD only groups (Table 2).

**Table 2.** Crude Incidence Rate of Fatal and Nonfatal Overdose Events During the 5-Year Follow-up Period

Characteristic	Nonfatal overdose		Fatal overdose	
	Total events, No.	No. of events/1000 person-years (95% CI)	Total deaths, No.	No. of deaths/1000 person-years (95% CI)
Total	3781	122.5 (118.6-126.5)	245	7.9 (7.0-9.0)
SUD diagnosis				
No OUD or STUD	2393	89.8 (86.3-93.5)	177	6.6 (5.7-7.7)
OUD only	397	312.4 (282.4-344.7)	17	13.4 (7.8-21.4)
STUD only	425	243.4 (220.8-267.7)	23	13.2 (8.3-19.8)
OUD and STUD^a^	566	467.5 (429.8-507.6)	28	23.1 (15.4-33.4)
Age group, y				
<30	1825	145.2 (138.6-152.0)	93	7.4 (6.0-9.1)
30-39	1178	130.7 (123.3-138.4)	77	8.5 (6.7-10.7)
≥40	778	83.9 (78.1-90.0)	75	8.1 (6.4-10.1)
Sex				
Male	3232	120.0 (115.9-124.2)	221	8.2 (7.2- 9.4)
Female	549	140.0 (128.5-152.2)	24	6.1 (3.9- 9.1)
Mental illness diagnosis^b^				
Yes	2366	187.8 (180.3-195.5)	125	9.9 (8.3-11.8)
No	1415	77.5 (73.5-81.6)	120	6.6 (5.4-7.9)
Elixhauser comorbidity index^c^				
None	3109	111.0 (107.1-114.9)	202	7.2 (6.2-8.3)
1	501	273.9 (250.4-299.0)	24	13.1 (8.4-19.5)
≥2	171	168.9 (144.5-196.2)	19	18.8 (11.3-29.3)
No. of incarceration events^d^				
1-2	637	43.2 (39.9-46.7)	81	5.5 (4.4-6.8)
3-4	593	96.0 (88.4-104.0)	52	8.4 (6.3-11.0)
5-7	696	151.6 (140.5-163.3)	44	9.6 (7.0-12.9)
≥8^e^	1855	346.3 (330.7-362.4)	68	12.7 (9.9-16.1)
Region of residence				
Interior	370	95.6 (86.1-105.9)	24	6.2 (4.0- 9.2)
Fraser	1391	146.6 (139.0-154.5)	86	9.1 (7.3-11.2)
Vancouver Coastal	1045	182.6 (171.7-194.0)	66	11.5 (8.9-14.7)
Island	613	145.8 (134.5-157.8)	44	10.5 (7.6-14.0)
Northern	327	112.2 (100.3-125.0)	21	7.2 (4.5-11.0)
Unspecified^f^	35	7.5 (5.2-10.4)	4	0.9 (0.2-2.2)

Abbreviations: OUD, opioid use disorder; STUD, stimulant use disorder; SUD, substance use disorder.

^a^ The crude incidence of fatal overdose among people with OUD and STUD (23.1 deaths per 100 000) represents a high burden of mortality. For context, this rate is 4 times higher than the incidence rate of mortality from motor vehicle accidents among British Columbians in 2021 (5.7 deaths per 100 000).

^b^ Mental illness includes anxiety, depression, or stress or adjustment disorder.

^c^ Excluding mental illness and substance use categories.

^d^ Occurred between 2010 and 2019.

^e^ Median, 3; mean, 4.9; range, 1-74; IQR, 1-6.

^f^ 1019 (15%) people had missing data on residential postal codes at baseline (2014), and therefore their region of residence could not be identified. Because the proportion is not small, they are reflected in an unspecified group so that persons with missing data on this variable could remain in the analysis. There was no missing data on other variables.

The unadjusted and adjusted Andersen Gill model (for recurrent NFOD events), and Fine and Gray model (for fatal overdose events) revealed a significantly higher risk of NFOD and fatal overdose events for people with OUD only, STUD only, and OUD and STUD compared with people with no OUD or STUD (Table 3). After adjusting for all covariates, the hazard of NFOD remained highest in the OUD and STUD group (HR, 2.45; 95% CI, 1.94-3.11), followed by the OUD only group (HR, 2.03; 95% CI, 1.57-2.62) and STUD only group (HR, 1.52; 95% CI, 1.20-1.92). In our study, the Fine and Gray competing risk model yielded very similar results to the results presented in the standard Cox model in sensitivity analysis (eTable 5 in the Supplement). This suggests that deaths from other causes had little influence on the effect estimates. This is likely because participants were young (mean [SD] age, 33.7 [10.7] years) and the risk of death from other causes was very small.

**Table 3.** Hazard Ratios for Fatal and Nonfatal Overdose in Relation to SUD Diagnosis^a^

SUD diagnosis	HR (95% CI)			
	Nonfatal overdose		Fatal overdose	
	Unadjusted	Adjusted^b^	Unadjusted	Adjusted^b^
No OUD or STUD	1[Reference]	1[Reference]	1[Reference]	1[Reference]
OUD only	3.37 (2.64-4.29)	2.03 (1.57-2.62)	1.99 (1.21-3.27)	1.58 (0.93-2.68)
STUD only	2.69 (2.13- 3.39)	1.52 (1.20-1.92)	1.96 (1.27-3.01)	1.46 (0.92-2.32)
OUD and STUD	5.15 (4.24-6.24)	2.45 (1.94-3.11)	3.45 (2.31-5.14)	2.39 (1.48-3.86)

Abbreviations: HR, hazard ratio; OUD, opioid use disorder; STUD, stimulant use disorder; SUD, substance use disorder.

^a^ The Andersen-Gill model was used for recurrent nonfatal overdose events, and the Fine and Gray competing risk model was used for fatal overdose events.

^b^ Adjusted HRs were controlled for age group, biological sex, mental illness, Elixhauser comorbidity index, social assistance, number of incarcerations during follow up, and location of residence (eTable 6 in the Supplement).

The OUD and STUD group also had the highest hazard of fatal overdose (HR, 2.39; 95% CI, 1.48-3.86). While the hazard of fatal overdose was elevated among people with OUD only and STUD only compared with the no OUD or STUD group in unadjusted analyses, this association did not hold in the adjusted analyses. Furthermore, there was little difference in the hazard of fatal overdose between the OUD only group (HR, 1.58; 95% CI, 0.93-2.68) and STUD only group (HR, 1.46; 95% CI, 0.92-2.32). The interaction term for age and SUD diagnosis was statistically significant (HR, 1.08; 95% CI, 1.02-1.16; *P* = .047) for the outcome of NFOD, suggesting that the effect of SUD diagnosis on NFOD depended on age. People in the 40 years or older age group had the highest hazard of NFOD of all SUD diagnosis groups (Figure). There was no evidence of interaction by sex (HR, 1.06; 95% CI, 0.92-1.22; *P* = .43) for NFOD nor for sex (HR, HR, 1.12; 95% CI, 0.80-1.56; *P* = .51) or age category (HR, 1.08; 95% CI, 0.93-1.20; *P* = .31) for fatal overdose (Figure).

**Figure.**
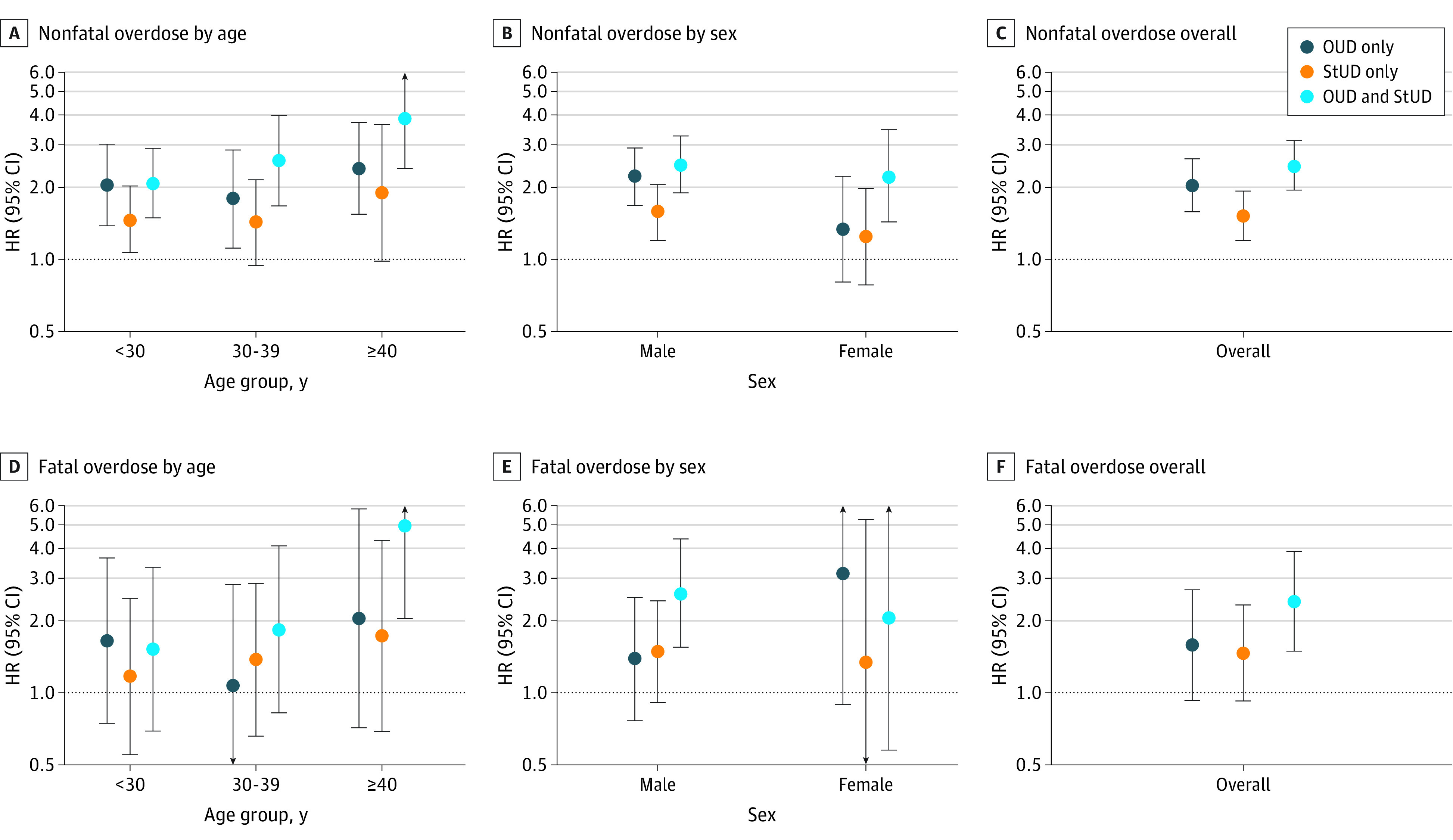
Fatal and Nonfatal Overdoses in British Columbia by Age and Sex HR indicates hazard ratio; OUD, opioid use disorder; STUD, stimulant use disorder.

## Discussion

People with histories of incarceration in BC face an elevated risk of overdose.^9,10^ Building on that research, we identified and quantified the risk facing people with histories of incarceration who have diagnoses of OUD, STUD, and both OUD and STUD as compared with people without these SUD diagnoses. In the context of rising illicit drug toxicity events across BC, particularly in the context of COVID-19,^19^ intervention for all groups is critical. Nevertheless, we found the incidence and hazard of fatal and nonfatal overdose was highest among people who had OUD and STUD diagnoses, highlighting the urgent need to scale up access to interventions to reduce overdose among this population.

Many people with concurrent OUD and STUD may be offered opioid agonist treatment (OAT) for OUD; however, stimulant use is known to interfere with OAT outcomes, such as retention.^20^ While evidence supporting interventions for STUD is still growing, in the context of SUD treatment more broadly, a range of interventions across a continuum of care is required to reach and engage people with a diversity of substance use patterns and treatment preferences.^21,22^ In the context of treatment for STUD, this could include psychosocial or pharmacological interventions. Recent studies^23,24^ have demonstrated that psychostimulants can be prescribed to support reductions in stimulant use among people with OUD and STUD. For example, patients prescribed dextroamphetamine in an OAT clinic in the Netherlands had significant reductions in cocaine use,^25^ and significant improvements in physical health, mental health, and social functioning.^26^ Similar positive outcomes have been identified in pilot programs in Canada.^27,28^ Furthermore, a 2021 systematic review^29^ found that contingency management was associated with psychostimulant abstinence among people with OUD and STUD when provided alongside OAT.

Among people with OUD and STUD, the potential effectiveness of both pharmacological and psychosocial treatments must be considered with attention to comorbidities. For example, attention-deficit hyperactivity disorder (ADHD) prevalence is estimated to be approximately 25% among people with SUDs^30^ and is elevated in justice-involved populations compared with the general population.^31^ Concurrent pharmacological treatment of ADHD can improve ADHD symptoms and reduce stimulant use.^32,33,34,35^ A randomized controlled trial^36^ in Sweden found that when prescribed 2-weeks before release and continued in community, methylphenidate reduced ADHD symptoms and relapse to substance use among people in prison with ADHD and SUD. Prisons are highly controlled environments, whereby medications, such as OAT, are widely prescribed (in BC), and psychostimulant medications for ADHD or STUD could be offered to support reductions in illicit stimulant use in the transition from prison to community. Furthermore, investments in peer-led interventions could be made to improve continuity of care, given the known effectiveness of such programs in building trust and connecting people to community services postrelease.^37,38^

In this study, we found a significant interaction between age and SUD diagnosis. Among people with both OUD and STUD, the oldest age group (≥40 years) had the highest hazard of fatal and nonfatal overdose. This could be partly explained by comorbidities, where 40% of people with OUD and STUD had a comorbidity compared with only approximately 7% of people with neither diagnosis. Cardiovascular disease (CVD) is common among patients with STUD^39,40^ and recent population-level analyses in BC show that overdose is associated with increased CVD risk.^41^ These data point to the need for CVD interventions for people with SUDs and to the need for STUD to be treated with long-term preventative interventions.^42^ There are interventions that could be more widely implemented immediately to address existing gaps in care. For example, stimulant use is a known risk factor for CVD; however, substance use is generally absent from CVD risk assessments and treatment plans.^42^ Furthermore, despite evidence that β-blockers are a safe and effective intervention for heart failure among people who use cocaine^43,44^ their use in this population is often avoided, due in part to stigma and misinformation.^45^

Consistent with prior studies,^11^ NFOD events made up approximately 94% of all overdose events in this study and the incidence of NFOD was more than 15 times that of fatal events. NFOD events have known cognitive and physical consequences attributed to loss of oxygen, including seizures, heart complications, neurological problems, and long-term brain injury,^46,47,48^ making it critical to reduce their prevalence. NFOD events also offer an opportunity to provide interventions when they do happen. For example, nearly half of people who died of overdose in this study had a prior NFOD event involving contact with health care (eg, hospital, emergency department, paramedic). This contact could serve as an important window of opportunity to provide interventions for OUD, STUD and associated comorbidities to people who desire them. For example, recent studies^49^ have pointed to the postoverdose period as a time when OAT can be initiated in emergency departments. Interventions for people with STUD can similarly be initiated through existing contacts with the care system postoverdose.

### Limitations

This study has a number of limitations that must be considered when interpreting findings. We rely on the use of *ICD-9* and *ICD-10* codes to identify SUDs, and thus our study does not represent people who may have an SUD but who are not in contact with health care. Of all fatal overdose events identified, more than 70% occurred among people with neither OUD nor STUD. This could reflect misclassification, whereby some cases of death could have occurred among people with OUD or STUD who were not in contact with health care, and thus were not identified as having OUD or STUD in our analysis. Nevertheless, recent reporting on illicit drug toxicity deaths in BC found that many people who died had not been previously engaged in SUD treatment, suggesting that the risk of overdose in BC extends beyond people with diagnosed SUDs.^50^ As such, cases of death in people with neither diagnosis could represent people who use drugs irregularly, and who do not have the same tolerance nor knowledge and skills^51,52^ to prevent or reduce overdose risk as people with more experience using drugs, including people with SUDs. Furthermore, some interventions meant to prevent overdose are available in BC for people with OUD that are not available to people without an OUD diagnosis. For example, OAT is available for people with an OUD diagnosis and has a known protective effect on overdose.^53^ This is an intervention that does not extend to offer protection from overdose for people with STUD only, or neither diagnosis.

## Conclusions

People who have been incarcerated are at elevated risk of overdose compared with the general population.^9^ This cohort study builds upon previous work and suggests that overdose risk is further elevated among people with histories of incarceration who have concurrent STUD and OUD diagnoses. As such, there is an urgent need to scale up interventions for this population. Patients’ existing contact with the health care system for OUD (eg, OAT) can be leveraged to expand services and to integrate STUD interventions into care. Medical comorbidities are common among people with OUD and STUD and must be identified and considered in treatment decision-making. In the context of frequent cycling between prison and community, continuity of care is critical to promote positive outcomes.

## Notes

Supplement. **eTable 1. **Outcome Measure: Nonfatal and Fatal Overdose Case Definitions **eTable 2.** Exposure: Type of Substance Use Disorder Diagnosis **eTable 3.** Comorbidities: *ICD-10* Codes Used in Elixhauser Comorbidity Categories **eTable 4.** Mental Illness Algorithm **eTable 5.** Hazard Ratios for Fatal Overdose in Relation to Each Characteristic of Interest Based on the Cox Proportional-Hazards Model **eTable 6.** Hazard Ratios for Drug Overdose in Relation to Each Characteristic of Interest
