# Supplementary material for: Association of Opioid and Stimulant Use Disorder Diagnoses With Fatal and Nonfatal Overdose Among People With a History of Incarceration
Source: JAMA Netw Open. 2022 Nov 23;5(11):e2243653. doi: 10.1001/jamanetworkopen.2022.43653 (PMC9685494; doi:10.1001/jamanetworkopen.2022.43653)
Supplement: Supplement. — eTable 1. Outcome Measure: Nonfatal and Fatal Overdose Case Definitions eTable 2. Exposure: Type of Substance Use Disorder Diagnosis eTable 3. Comorbidities: ICD-10 Codes Used in Elixhauser Comorbidity Categories eTable 4. Mental Illness Algorithm eTable 5. Hazard Ratios for Fatal Overdose in Relation to Each Characteristic of Interest Based on the Cox Proportional-Hazards Model eTable 6. Hazard Ratios for Drug Overdose in Relation to Each Characteristic of Interest [file jamanetwopen-e2243653-s001.pdf]

Supplemental Online Content

Palis H, Gan W, Xavier C, et al. Association of opioid and stimulant use disorder diagnoses with fatal and nonfatal overdose among people with a history of incarceration. *JAMA Netw Open*. 2022;5(11):e2243653. doi:10.1001/jamanetworkopen.2022.43653

eTable 1. Outcome Measure: Nonfatal and Fatal Overdose Case Definitions

eTable 2. Exposure: Type of Substance Use Disorder Diagnosis

eTable 3. Comorbidities: *ICD-10* Codes Used in Elixhauser Comorbidity Categories

eTable 4. Mental Illness Algorithm

eTable 5. Hazard Ratios for Fatal Overdose in Relation to Each Characteristic of Interest Based on the Cox Proportional-Hazards Model

eTable 6. Hazard Ratios for Drug Overdose in Relation to Each Characteristic of Interest

This supplemental material has been provided by the authors to give readers additional information about their work.

© 2022 Palis H et al. *JAMA Network Open.*

eTable 1. Outcome Measure: Fatal and Nonfatal Overdose Case Definitions

**NON-FATAL OVERDOSE**	
**BC Emergency Health Services: Patient Care Information System**	Naloxone administered by paramedics OR Impression code: Recreational Drug OD AND CARD in (9,23,26,31)*, OR Impression code: Cardiac arrested: treated and CARD 23
**BC Emergency Health Services: Siren**	Naloxone administered OR Impression code: 'Opioid-related', 'opioid-related/OD', OR Impression code: Cardiac arrest and CARD 23*
**Drug and Poison Information Centre**	Any call with a code related to drug overdose/ poisoning
**Enhanced Emergency Department**	Case-based reports by Emergency Departments by participating health authority
**Medical Services Plan, Discharge Abstract Database, National Ambulatory Care Reporting System**	Case defined by ICD version 9 and 10 (e.g., 965.0-poisoning by opiates and related narcotics; E850.0-accidental poisoning by opiates and related narcotics) OR *CARD 9: Cardiac or respiratory arrest/death; CARD 23: Overdose/poisoning (ingestion); CARD 26: Sick; CARD 31: Unconscious
**FATAL OVERDOSE**	
**BC Coroners Service**	Open investigations (toxicology pending) and closed drug overdose deaths
**Vital Statistics Deaths**	Case defined by ICD version 9 and 10 (e.g., 965.0-poisoning by opiates and related narcotics; E850.0-accidental poisoning by opiates and related narcotics) OR *CARD 9: Cardiac or respiratory arrest/death; CARD 23: Overdose/poisoning (ingestion); CARD 26: Sick; CARD 31: Unconscious
**Drug-related overdose algorithm**	If death date from other administrative databases (e.g., client roster) lies between the start date and end date of an overdose episode, then this overdose episode is defined as a fatal overdose. In these cases, please use the overdose episode start date as the date of the fatal overdose

© 2022 Palis H et al. *JAMA Network Open.*

eTable 2. Exposure: Type of Substance Use Disorder Diagnosis

Diagnosis	ICD9 codes	ICD10 codes	Algorithm
**Substance use disorder**			
Stimulant use disorder	3042, 3052, 3044, 3057	F14, F15	Either: 2 family physician visits or 1 hospitalization in 1 year
Opioid use disorder	3040, 3047, 3055	F11	Either: 2 family physician visits or 1 hospitalization in 1 year

© 2022 Palis H et al. *JAMA Network Open.*

eTable 3. Comorbidities: *ICD-10* codes used in Elixhauser comorbidity categories

Category	ICD-10 codes
1. Congestive heart failure	I099, I110, I130, I193, I255, I420, I425, I426, I427, I428, I429, I43, I50, P290
2. Cardiac arrhythmia	I441, I442, I443, I456, I459, I47, I48, I49, R000, R001, R008, T821, Z450, Z950
3. Valvular disease	A520, I05, I06, I07, I08, I091, I098, I34, I35, I36, I37, I38, I39, Q231, Q232, Q233, Z952, Z953, Z954
4. Pulmonary circulation disorders	I26, I27, I280, I288, I289
5. Peripheral vascular disorders	I70, I71, I731, I738, I739, I771, I790, I792, K551, K558, K559, Z958, Z959
6. Hypertension uncomplicated	I10
7. Hypertension complicated	I11, I12, I13, I15
8. Paralysis	G041, G114, G801, G802, G81, G82, G830, G831, G832, G833, G834, G839
9. Other neurological disorders	G10, G11, G12, G13, G20, G21, G22, G254, G255, G312, G318, G319, G32, G35, G36, G37, G40, G41, G931, G934, R470, R56
10. Chronic pulmonary disease	I278, I279, J40, J41, J43, J44, J45, J46, J47, J60, J61, J62, J63, J64, J65, J66, J67, J684, J701, J703
11. Diabetes uncomplicated	E100, E101, E109, E110, E111, E119, E120, E121, E129, E130, E131, E139, E140, E141, E149
12. Diabetes complicated	E10-E0E108, E112-E118, E122-E128, E132-E138, E14-E148
13. Hypothyroidism	E00-E03, E890
14. Renal Failure	I120, I131, N18, N19, N250, Z490, Z491, Z492, Z940, Z992
15. Liver disease	B18, I85, I865, I982, K70, K711, K713, K714, K715, K717, K72, K73, K74, K760, K762-K769, Z94
16. Peptic ulcer excluding bleeding	K257, K259, K267, K269, K277, K279, K287, K289
17. HIV/AIDS	B20, B21, B22, B24
18. Lymphoma	C81-C85, C88, C96, C900, C902
19. Metastic cancer	C77-C80
20. Solid tumor without metastasis	C00-C26, C30-C34, C37-C41, C43, C45-C58, C60-C76, C97
21. Rheumatoid arthritis/collagen	L940, L941, L943, M05, M06, M08, M120, M123, M30, M310, M311, M312, M313, M32, M33, M34, M35, M45, M461, M468, M469
22. Coagulopathy	D65, D66, D67, D68, D691, D693, D694, D695, D696
23. Obesity	E66
24. Weight loss	E40-E46, R634, R64
25. Fluid and electrolyte disorders	E222, E86, E87
26. Blood loss anemia	D500
27. Deficiency anemia	D508, D509, D51, D52, D53

Based on ICD-10 codes categorised by Quan et al[1]

[1] Quan, H., Sundararajan, V., Halfon, P., Fong, A., Burnand, B., Luthi, J. C., ... & Ghali, W. A. (2005). Coding algorithms for defining comorbidities in ICD-9-CM and ICD-10 administrative data. *Medical care*, 1130-1139

© 2022 Palis H et al. *JAMA Network Open.*

eTable 4. Mental Illness Algorithm

Diagnosis	ICD9 codes	ICD10 codes	Algorithm
**Mental illness**			
Depression	300.4<br>311<br>50B (also requires another code to qualify)	F32<br>F33<br>F34.1	Either: 2 family physician visits or 1 hospitalization in 1 year
Anxiety	300 (excluding 300.4)<br>50B (also requires another code to qualify)	F40<br>F41	Either: 2 family physician visits or 1 hospitalization in 1 year
Stress and adjustment disorder	308<br>309	F43	Either: 2 family physician visits or 1 hospitalization in 1 year
Schizophrenia	295<br>297<br>298	F20<br>F21<br>F22<br>F23<br>F24<br>F25<br>F28<br>F29	Either: 2 family physician visits or 1 hospitalization in 1 year
Bipolar disorder	296	F30<br>F31<br>F34 (excluding F34.1)<br>F38<br>F39	Either: 2 family physician visits or 1 hospitalization in 1 year

© 2022 Palis H et al. *JAMA Network Open.*

eTable 5. Hazard ratios for fatal overdose in relation to each characteristic of interest based on the Cox proportional-hazards model

Characteristic	Fatal overdose	
	**Unadjusted HR (95% CI)**	**Adjusted HR (95% CI)[a]**
SUD diagnosis		
Neither	Reference (1.00)	Reference (1.00)
OUD only	2.00 (1.22-3.29)	1.58 (0.95-2.64)
StUD only	1.98 (1.28-3.06)	1.47 (0.93-2.34)
OUD & StUD	3.46 (2.32-5.16)	2.37 (1.50-3.75)
Age group, years		
< 30	Reference (1.00)	Reference (1.00)
30 - 39	1.16 (0.86-1.57)	1.13 (0.84-1.54)
≥ 40	1.10 (0.81-1.49)	1.15 (0.83-1.57)
Sex		
Females	Reference (1.00)	Reference (1.00)
Males	1.33 (0.88-2.03)	1.65 (1.07-2.55)
Mental illness diagnosis		
No	Reference (1.00)	Reference (1.00)
Yes	1.51 (1.17-1.94)	1.04 (0.79-1.36)
Elixhauser comorbidity index[b]		
None	Reference (1.00)	Reference (1.00)
1	1.82 (1.19-2.78)	1.23 (0.78-1.91)
≥ 2	2.61 (1.63-4.18)	1.79 (1.09 -2.95)
Number of incarceration[c]		
1 - 2	Reference (1.00)	Reference (1.00)
3 - 4	1.52 (1.07-2.15)	1.65 (1.16-2.35)
5 - 7	1.71 (1.18-2.47)	1.76 (1.21-2.55)
≥ 8	2.25 (1.62-3.11)	2.02 (1.44-2.83)
Region of residence		
Interior	Reference (1.00)	Reference (1.00)
Fraser	1.47 (0.93-2.30)	1.38 (0.88-2.18)
Vancouver Coastal	1.87 (1.17-2.98)	1.54 (0.96-2.48)
Island	1.69 (1.03-2.78)	1.64 (1.00-2.70)

© 2022 Palis H et al. *JAMA Network Open.*

Characteristic	Fatal overdose	
	**Unadjusted HR (95% CI)**	**Adjusted HR (95% CI)[a]**
Northern	1.16 (0.65-2.08)	1.13 (0.63-2.04)
Unspecified	0.14 (0.05-0.40)	0.14 (0.05-0.40)

CI = confidence interval; HR = hazard ratio; OUD = opioid use disorder; StUD = stimulant use disorder; SUD = substance use disorder.

[a] Adjusted HRs were controlled for all other variables in the table.

[b] Excluding mental illness and substance use categories.

[c] Occurred between 2010 and 2019.

© 2022 Palis H et al. *JAMA Network Open.*

eTable 6. Hazard Ratios for Drug Overdose in Relation to Each Characteristic of Interest[a]

Characteristic	Non-fatal overdose		Fatal overdose	
	**Unadjusted HR (95% CI)**	**Adjusted HR (95% CI)[b]**	**Unadjusted HR (95% CI)**	**Adjusted HR (95% CI)[b]**
SUD diagnosis				
Neither	Reference (1.00)	Reference (1.00)	Reference (1.00)	Reference (1.00)
OUD only	3.37 (2.64-4.29)	2.03 (1.57-2.62)	1.99 (1.21-3.27)	1.58 (0.93-2.68)
StUD only	2.69 (2.13-3.39)	1.52 (1.20-1.92)	1.96 (1.27-3.01)	1.46 (0.92-2.32)
OUD & StUD	5.15 (4.24-6.24)	2.45 (1.94-3.11)	3.45 (2.31-5.14)	2.39 (1.48-3.86)
Age group, years				
< 30	Reference (1.00)	Reference (1.00)	Reference (1.00)	Reference (1.00)
30 - 39	0.92 (0.79-1.08)	0.91 (0.78-1.06)	1.15 (0.85-1.56)	1.13 (0.84-1.53)
≥ 40	0.61 (0.51-0.73)	0.71 (0.60-0.84)	1.08 (0.80-1.46)	1.12 (0.81-1.54)
Sex				
Females	Reference (1.00)	Reference (1.00)	Reference (1.00)	Reference (1.00)
Males	0.82 (0.69-0.98)	1.09 (0.92-1.30)	1.34 (0.88-2.04)	1.65 (1.07-2.54)
Mental illness diagnosis				
No	Reference (1.00)	Reference (1.00)	Reference (1.00)	Reference (1.00)
Yes	2.42 (2.10-2.78)	1.49 (1.28-1.73)	1.50 (1.17-1.93)	1.04 (0.78-1.37)
Elixhauser comorbidity index[c]				
None	Reference (1.00)	Reference (1.00)	Reference (1.00)	Reference (1.00)
1	2.47 (1.98-3.09)	1.46 (1.15 - 1.86)	1.77 (1.16 - 2.71)	1.20 (0.76 - 1.90)
≥ 2	1.57 (1.14-2.17)	1.03 (0.75 - 1.42)	2.38 (1.49 - 3.80)	1.66 (1.01 - 2.73)
Number of incarceration[d]				
1 - 2	Reference (1.00)	Reference (1.00)	Reference (1.00)	Reference (1.00)
3 - 4	1.84 (1.55-2.19)	1.92 (1.61-2.28)	1.52 (1.07-2.15)	1.66 (1.17-2.35)
5 - 7	2.76 (2.32-3.30)	2.78 (2.33-3.31)	1.71 (1.19-2.47)	1.75 (1.20-2.54)
≥ 8	4.72 (4.01-5.56)	4.21 (3.57-4.98)	2.26 (1.64-3.11)	2.03 (1.44-2.85)
Region of residence				
Interior	Reference (1.00)	Reference (1.00)	Reference (1.00)	Reference (1.00)
Fraser	0.29 (0.23-0.38)	0.17 (0.13-0.22)	1.48 (0.94-2.32)	1.39 (0.88-2.19)
Vancouver Coastal	1.95 (1.53-2.50)	1.35 (1.06-1.73)	1.88 (1.18-2.99)	1.55 (0.97-2.48)
Island	1.54 (1.17-2.02)	1.40 (1.08-1.82)	1.70 (1.03-2.79)	1.65 (1.00-2.71)
Northern	1.17 (0.84-1.61)	1.07 (0.78-1.46)	1.16 (0.65-2.09)	1.13 (0.63-2.04)

© 2022 Palis H et al. *JAMA Network Open.*

Characteristic	Non-fatal overdose		Fatal overdose	
	**Unadjusted HR (95% CI)**	**Adjusted HR (95% CI)[b]**	**Unadjusted HR (95% CI)**	**Adjusted HR (95% CI)[b]**
Unspecified	0.08 (0.04-0.14)	0.08 (0.04-0.14)	0.14 (0.05-0.41)	0.14 (0.05-0.40)

CI = confidence interval; HR = hazard ratio; OUD = opioid use disorder; StUD = stimulant use disorder; SUD = substance use disorder.

[a] The Andersen-Gill model was used for recurrent non-fatal overdose events, and the Fine & Gray competing risk model was used for fatal overdose events.

[b] Adjusted HRs were controlled for all other variables in the table.

[c] Excluding mental illness and substance use categories.

[d] Occurred between 2010 and 2019.

© 2022 Palis H et al. *JAMA Network Open.*
